# Discounting seems the most toxic dimension of invalidation in fibromyalgia: a cross-sectional analysis

**DOI:** 10.1007/s00296-025-05850-2

**Published:** 2025-04-15

**Authors:** Heidi Willemse, Johanna E. Vriezekolk, Rinie Geenen

**Affiliations:** 1 Department of Psychology, Utrecht University, P.O. Box 80140, Utrecht, 3508 TC The Netherlands; 2 Department of Research, Sint Maartenskliniek, P.O. Box 9011, Nijmegen, 6500 GM The Netherlands

**Keywords:** Anxiety, Depression, Fibromyalgia, Invalidation, Physical health, Social rejection, Surveys and questionnaires

## Abstract

Invalidation, both discounting (overt negative social responses) and lack of understanding (absence of positive social responses), is a common problem in fibromyalgia. The ‘Fibromyalgia Imbalance of Threat and Soothing Systems’ (FITSS) model indicates that different neuropsychological processes may underlie these two components of invalidation. Guided by this model, the aim of the current study was to clarify the differentiation between these two components of invalidation by examining their association with fibromyalgia severity, anxiety, and depression. This cross-sectional study included the Illness Invalidation Inventory (3*I), the Fibromyalgia Impact Questionnaire (FIQ), and the Hospital and Depression Scale (HADS). Demographics of the 280 respondents with fibromyalgia were: mean age 42.6 ± 11.8 yrs., 95% female, mean FIQ score 59.1 ± 15,5, possible or probable cases of anxiety and depression, 49% and 42%, respectively. Regression analyses revealed that discounting was associated with severity of fibromyalgia (t = 4.10, β = 0.34, p <.001), anxiety (t = 3.50, β = 0.29, p <.001) and depression (t = 3.64, β = 0.30, p <.001) symptoms. Neither lack of understanding (-1.62 ≤ t ≤.10, -0.13 ≤ β ≤ 0.01, p ≥ 0.11) nor the interaction of discounting and lack of understanding (-0.19 ≤ t ≤ 1.10, -0.01 ≤ β ≤ 0.07, p ≥ 0.27) was related to any of the outcome variables. The total model accounted for 8.8%, 5.3%, and 8.3% (adjusted R^2^) of variance in fibromyalgia severity, anxiety, and depression, respectively. In relation to both mental and physical health, discounting seems the most toxic dimension of invalidation in fibromyalgia. This suggests that overt negative responses should get attention in its management, especially in more severe fibromyalgia. Both people with fibromyalgia and people in their environment have a role in reducing invalidation.

**Supplementary Information:**

The online version contains supplementary material available at 10.1007/s00296-025-05850-2.

## Introduction

Fibromyalgia is characterized by widespread pain and other symptoms such as fatigue, unrefreshed sleep, and cognitive problems [1]. Central sensitization has been suggested to be the neurophysiological mechanism underlying fibromyalgia [2]. Nevertheless, there is a lot of uncertainty about the etiological and maintaining factors of fibromyalgia. The recently introduced FITSS model (Fibromyalgia: Imbalance of Threat and Soothing Systems) is an attempt to integrate essential psychosocial and neuropsychological mechanisms in fibromyalgia [3]. The model includes two complex networks of neuroanatomic structures involved in affect regulation, the threat and soothing systems as defined by Gilbert [4, 5]. The threat system is programmed to detect and evaluate impending or anticipated threats and promote defensive actions, while the soothing system is linked to prosocial and affiliative behaviors as well as positive affect states. It has been suggested that an imbalance in the threat and soothing systems, as expressed by a hyperactive threat system and a hypoactive soothing system, may affect the severity of fibromyalgia [3]. Besides and in interaction with biological factors, psychosocial factors are assumed to be able to activate the threat and soothing systems and amplify the severity of fibromyalgia. Neuropsychological studies suggest that negative psychosocial factors, such as social rejection, can contribute to symptom amplification by activating the sensitized brain [6–9] and studies tentatively indicate that positive psychosocial factors (such as social support) might mitigate symptoms by dampening the sensitized brain [6, 10, 11]. Further understanding of the link between fibromyalgia severity and psychosocial processes such as social rejection and support may provide clues on potential ways to improve life for persons with fibromyalgia.

In our previous work in patients with fibromyalgia and rheumatoid arthritis, we distinguished two types of social responses, called invalidation: discounting (overt negative social responses) and lack of understanding (absence of positive social responses) [12]. Discounting represents disbelieving, admonishing, dismissing inability to work, not acknowledging symptom fluctuations, and offering unusable advice. Lack of understanding comprises not recognizing, not comprehending, and not emotionally supporting the person with fibromyalgia [13]. On a neurophysiological level, discounting may be assumed to activate the threat system and amplify pain and other symptoms; while understanding or social support activates the soothing system and mitigates pain [3]. This suggests that discounting and (lack of) understanding are additively related to the health status of people with fibromyalgia.

Our previous work in patients with various rheumatic disease suggested the usefulness of differentiating between discounting and lack of understanding. While taking account of social support, both discounting and lack of understanding were associated with mental health, but only invalidation was significantly associated with physical health [14]. The result suggests that improving health of patients with rheumatic diseases requires the consideration of both discounting and lack of understanding. Several other studies in people with fibromyalgia [15–17] and other chronic pain conditions [18–20] confirmed that discounting and lack of understanding are additively associated with various outcome measures. Discounting was also stronger than lack of understanding associated with reduced physical health [15], symptom severity [16], and visits to physicians [17]. One study did not observe distinctive patters of correlation of discounting and lack of understanding with mental and physical health [21]. However, with that study as the only exception, discounting seems the most toxic dimension of invalidation in persons with fibromyalgia, especially where it concerns physical health.

Additionally, the two dimensions of invalidation might be differently associated with anxiety and depression symptoms. Both anxiety and depression are prevalent problems in fibromyalgia [22]. Previous research in chronic widespread pain [20] and other groups than fibromyalgia [22, 23], found a positive association of invalidation with anxiety and depression symptoms [21, 23, 24]. However, none of these studies simultaneously examined the association of both discounting and lack of understanding with anxiety and depression symptoms. Although anxiety and depression are interrelated, they also reflect different processes. Anxiety symptoms appear more closely associated with pain than depression symptoms [25]. Anxiety more clearly reflects an enhanced threat system including threat from others. This might imply that anxiety is stronger associated with discounting than depression. To fill this knowledge gap and to get more insight into the need for a tailored focus in therapy, the mutual associations of discounting and lack of understanding with anxiety and depression symptoms should be examined.

Therefore, guided by the FITSS model, the current study aims to clarify whether and to what extent invalidation, both discounting and (lack of) understanding, are associated with fibromyalgia severity, anxiety and depression symptoms. It is expected that discounting and (lack of) understanding are additively associated with fibromyalgia severity, with discounting being stronger related to physical health and anxiety than lack of understanding.Findings of this study will indicate whether it is useful to differentiate between discounting and (lack of) understanding and for which outcomes of fibromyalgia specifically, advancing both research and therapy.

## Methods

### Procedure and participants

This cross-sectional analysis of baseline self-report data is part of a longitudinal cohort study on psychological and psychosocial outcomes of fibromyalgia. Recruitment and data collection were between December 2011 and May 2013. Participants were persons with fibromyalgia referred to the Sint Maartenskliniek, rheumatology outpatient clinic, locations Nijmegen and Woerden, the Netherlands. Consecutive patients were included in the cohort after being classified as having fibromyalgia by certified rheumatologists. They were 18 years or older at time of diagnosis, were able to read and write Dutch language, and gave informed consent. The study adhered to the recommendations on reporting survey studies [26]. Questionnaires were distributed either by postal mail (paper-and-pencil) or via an email survey link (NETQ platform), depending on participants’ preferred mode. Survey reminders were sent two weeks after the baseline assessment. Data were anonymized before being entered in the data filethat was used in analysis. The study was performed in accordance with the ethical standards of the 1964 Declaration of Helsinki and its amendments. The Institutional Review Board of the Radboud University Medical Centre, Nijmegen concluded that the Medical Research Involving Human Subjects Act did not apply to this study (protocol number: 2011/271). All 280 participants completed the relevant measurements and were included in analyses.

### Instruments

The data used in the current study included three validated questionnaires and three items about demographic characteristics, namely: age, sex, and education. All included questionnaires are freely available questionnaires that can be used for research purposes if a reference to the original publication is added.

#### Invalidation

To measure invalidation, the Illness Invalidation Inventory (3*I) was used [12]. This measurement consists of eight items covering the two dimensions: lack of understanding (three items) and discounting (five items). This self-rated survey measures the occurrence of invalidation by five different sources: spouse, family, medical professionals, work environment, and social services. When a source category did not apply (e.g., because the person was not in employment), this category could be skipped. The items are scored using a 5-point Likert scale ranging from 1 (never) to 5 (very often). This measure has shown good reliability and validity [12]. In the current sample, the internal consistency was good with Cronbach’s alpha’s > 0.81 for all sources. In the present study, the mean scores across the five sources for discounting and lack of understanding were calculated when at least three source subscales were present [14]. Higher scores indicate more invalidation.

#### Severity of fibromyalgia

To measure fibromyalgia severity, the Fibromyalgia Impact Questionnaire (FIQ), a self-rated 10-item measurement for assessing health status in fibromyalgia was used [27]. The Dutch version has shown good reliability and validity [28]. If one or two of the 10 items was left blank, the scores obtained were summed and divided by the number of questions answered. Total scores range from 0 to 100. Higher scores indicate a greater impact of fibromyalgia on the individual. The reliability in the current study was good with a Cronbach’s alpha of 0.79.

#### Anxiety and depression

Anxiety and depression symptoms were measured using the self-rated 14-item Hospital Anxiety and Depression Scale (HADS) [29]. This measure was developed for chronically ill populations and excludes bodily symptoms (e.g., sleep problems, fatigue) that may be a consequence of a chronic illness. Both anxiety and depression consist of 7 items, using a 4-point Likert scale ranging from 0 to 3 with higher scores indicating more anxious/depression symptoms (after reverse coding of 8 items) [29]. Besides a continuous score, clinical classification schemes have been used to categorize the HADS scores, resulting in the following suggested cutoff scores: 0–7 = no depression/anxiety, 8–10 = possible anxiety/depression, 11–21 = probable anxiety/depression [29]. This measure has shown good reliability and validity in previous research [30]. The reliability in the current study was good with Cronbach’s alphas of 0.81 and 0.82 for anxiety and depression, respectively.

### Statistical analyses

Analyses were done with the Statistical Package for Social Sciences (SPSS Version 27.0, IBM Corp., Armonk, NY, USA). Two-sided *p*-values < 0.05 were considered statistically significant. Categorical data were presented as numbers and percentages, and continuous variables as means (SD). Score distributions of the six independent and dependent continuous variables were checked [31].

Univariate correlations between variables were evaluated by Pearson correlation coefficients. Ancillary univariate correlations were calculated for the subscales of the 3*I (spouse, family, medical professionals, work environment, and social services) to examine whether observed results reflected particular sources of invalidation.

Multivariable regression analyses were performed to examine the association of invalidation (discounting, lack of understanding, and the interaction of discounting x lack of understanding) with fibromyalgia severity, anxiety, and depression. Variables were centered before interactions were computed. Violations of multicollinearity and assumptions (linearity, homoscedasticity, normality) were checked [32]. The demographic variables that had a statistically significant correlation with any of the three dependent variables were included in all analyses.

## Results

### Participant characteristics

Table 1 shows the characteristics of the participants. The majority of the sample was female and received a middle to high level of education. According to the HADS criteria, 23% of the participants were classified as possible cases of anxiety, and 26% as probable cases. Regarding the depression subscale, 25% of the participants were classified as possible cases of depression, and 17% as probable cases. Compared to other fibromyalgia samples, the current sample reported similar scores of fibromyalgia severity [e.g., 33, 34] and invalidation [16, 35], but relatively low scores of anxiety and depression [e.g., 36, 37]. However, there are no normative data to ensure the relevance of the differences. Skewness and kurtosis of the six independent and dependent continuous variables indicated normal score distributions.

**Table 1:** Characteristics of the study sample (*N* = 280)

	*n*	%	M	SD	Range	Missing values
Sex						0
*Female*	267	95				
*Male*	13	5				
Age in years			42.6	11.8	18–73	0
Education Level ^a^						15
*Low*	53	19				
*Middle-High*	212	76				
Fibromyalgia severity (FIQ) ^b^			59.1	15.5	10–92	0
Invalidation (3*I) ^c^						19
*Discounting*			2.2	0.7	1-4.2	
*Lack of Understanding*			2.6	0.6	1-4.5	
Anxiety (HADS) ^d^			7.9	4.2	0–21	0
*Normal*	139	50				
*Possible/probable anxiety*	141	50				
Depression (HADS) ^d^			7.0	3.8	0–19	0
*Normal*	162	58				
*Possible/probable depression*	118	42				

^a^ = Education level: low = no education, lower primary school. lower secondary education

^b^ = FIQ = Fibromyalgia Impact Questionnaire

^c^ = 3*I = Illness Invalidation Inventory

^d^ = HADS = Hospital Anxiety and Depression Scale

### Associations between variables

Table 2 shows the univariate correlations between all variables. The correlation between discounting and lack of understanding was strong (*r* = 0.64). The three outcome variables were more often associated with discounting than lack of understanding. Small significant correlations were observed between discounting and fibromyalgia severity, anxiety, and depression as well as between lack of understanding and depression. Of the covariates, only a younger age showed a weak significant correlation with fibromyalgia severity and anxiety.

**Table 2:** Correlations between invalidation (discounting and lack of understanding), severity of fibromyalgia, anxiety, and depression

	1	2	3	4	5
1. Invalidation: discounting (3*I)	-				
2. Invalidation: lack of understanding (3*I)	0.64^*^	-			
3. Fibromyalgia severity (FIQ)	0.29^**^	0.10	-		
4. Anxiety (HADS)	0.24^**^	0.09	0.48^**^	-	
5. Depression (HADS)	0.29^**^	0.19^**^	0.49^**^	0.62^**^	-
*6. Age*	-0.22^**^	-0.11	-0.12^*^	-0.12^*^	0.03
*7. Sex*	-0.09	-0.06	-0.07	0.01	-0.07
*8. Education*	0.18^**^	0.14^*^	-0.08	-0.05	-0.04

^*^p < 0.05; ^**^p < 0.01

3*I = Illness Invalidation Inventory, FIQ = Fibromyalgia Impact Questionnaire, HADS = Hospital Anxiety and Depression Scale

Results of the ancillary analyses regarding source-specific invalidation scores are shown in Table S1 of the supplementary file. The range of correlations for specific sources reflected that the three outcome variables were more clearly associated with discounting (0.07 ≤ *r* ≤ 0.33) than lack of understanding (-0.03 ≤ *r* ≤ 0.24).

### Association of invalidation with fibromyalgia severity

Table 3 shows the results of regression analyses of the associations of fibromyalgia severity, anxiety, and depression with the two invalidation dimensions (discounting and lack of understanding) and their interaction. No violations of multicollinearity and assumptions occurred: VIFs varied from 1.07 to 1.94, residuals were symmetrically distributed, and normal probability plots showed scores close to the diagonal. The analyses indicated that of the two invalidation dimensions, discounting, but not lack of understanding, was associated with the three outcome variables. The interactions of discounting and lack of understanding were not significantly associated with any outcome variable. The total model accounted for 8.8% of variance in fibromyalgia severity, 5.3% of anxiety, and 8.3% of depression (adjusted R^2^). We repeated the analyses of fibromyalgia severity with anxiety and depression symptoms excluded from the FIQ score, but this did not change the results. Significance levels were *p* = 0.44 (age), *p* < 0.001 (discounting), *p* = 0.12 (lack of understanding), and *p* = 0.21 (the interaction discounting x lack of understanding).

**Table 3:** Results of multivariable regression analyses examining the associations of severity of fibromyalgia, anxiety, and depression with age, Discounting and Lack of understanding, and the interaction between Discounting and Lack of understanding

Variables	Fibromyalgia severity			Anxiety			Depression		
	β	t	*p*	β	t	*p*	β	t	*p*
Age	-0.06	-1.05	0.30	-0.07	-1.04	0.30	0.10	1.55	0.12
Discounting	0.34	4.10	< 0.001	0.29	3.50	< 0.001	0.30	3.64	< 0.001
Lack of understanding	-0.13	-1.62	0.11	-0.11	-1.35	0.18	0.01	0.10	0.92
Discounting x Lack of understanding	0.07	1.10	0.27	-0.01	-0.19	0.85	0.04	0.60	0.55

## Discussion

This study aimed to clarify whether and to what extent discounting and lack of understanding are associated with severity of fibromyalgia, anxiety, and depression. Persons with fibromyalgia with higher discounting scores reported more severe fibromyalgia and anxiety and depression symptoms. The strength of associations was small. Lack of understanding only showed a significant and small correlation with depression symptoms; this association disappeared when account was taken of discounting scores. These results did confirm our hypothesis that discounting is stronger than lack of understanding related to severity of fibromyalgia and anxiety. Yet, our expectation that discounting and (lack of) understanding would be additively associated with fibromyalgia severity was not confirmed.

Correlations of discounting with fibromyalgia severity, anxiety and depression were significant but small. This is not unexpected since it is only one variable in the network of potentially relevant variables. The severity of fibromyalgia can be influenced by biological, psychological, and social variables. These three classes of variables are often mutually related, and none of them has such a uniform influence that it is considered primary in treatment of fibromyalgia. For instance, the role of central sensitization could imply that pharmacologically altering neurotransmission involved in pain transmission might be effective. However, effects of pharmacological treatment, e.g., serotonin and noradrenaline reuptake inhibitors (SNRIs), are not strong, and are only observed in part of the patients [38–40]. There are also numerous studies showing psychological distress in fibromyalgia. With respect to potential psychological influencing factors, the disposition towards negative affectivity, e.g., neuroticism and type D personality, has been shown prevalent in fibromyalgia [41–43]. Although it is not clear whether this is a cause or consequence of fibromyalgia, or both, it makes sense to focus on management of psychological distress if it is high. The same holds for management of social variables. The main results of the current study, i.e., the observed association of discounting with fibromyalgia severity, anxiety and depression, must be seen as one of many variables that can play a significant role in some individual patients but not in others [44]. If an intake or questionnaire assessment shows such a role, it could be a focus in tailored treatment [40].

The current results confirm previous studies observing that discounting is stronger related to physical pain and severity of fibromyalgia than lack of understanding [14–16, 18–20]. This finding is also in line with the notion that social threats sensitize neural networks in the brain that play a role in amplification of pain [6, 45]. However, there may be many more explanations. It is, for instance, possible that more severe symptoms make people vulnerable to experience danger such as negative social reactions in their environment or that both vulnerability for discounting and more severe symptoms have to do with neurophysiological changes early in life, e.g., due to childhood maltreatment such as abuse or neglect [46] and disturbed attachment experiences [47, 48]. Our study does not give insight into these mechanisms, but it confirms that negative overt social reactions (discounting) are stronger related to physical health of people with fibromyalgia than the absence of positive social reactions (lack of understanding).

A distinguishing feature of our study was the examination of the association between the two dimensions of invalidation with anxiety and depression. Guided by the FITSS model [3], we expected that anxiety, because it is conceptually related more to the threat system, would be stronger associated with discounting than depression, but this hypothesis was refuted. Multiple studies offer accounts of the buffering effects of social support against anxiety and depression. It appears that (lack of) understanding, which especially reflects (lack of) acknowledgement of the illness and person, is different from social support. One of the possible inferences from our cross-sectional observation as contrasted with social support research is that discounting is harmful for anxiety and depression, not getting understanding is less harmful, and social support may protect against psychological distress. The lack of visible limitations and evidence of illness or injury in fibromyalgia make it difficult for others to understand the condition. Invalidation as perceived by a person may partly reflect this response of others. Reduction of invalidation should be a focus for health policy makers, patient associations, societal organizations and people near the patient. They should not deny the existence of symptoms that cannot be observed and they should not lecture, patronize, or overprotect the person. Instead, they should listen, try to understand, and acknowledge the illness and the person, and offer practical help and emotional support.

Whether invalidation occurs and how it is perceived by the person with fibromyalgia will also partly depend on the person. The intercorrelations between the five sources of invalidation as shown in the supplementary file support this theory: persons who report more invalidation from one source tend to perceive more invalidation from several other sources as well. Thus, in interaction with the actual environmental, invalidation also depends on the perception and skills of the person. Confronted with invalidation, the person can try to change this situation by offering the other person information about the disease or by making clear what invalidation means and asking for understanding, and if the situation cannot be changed to accept and tone down the consequences of invalidation [49]. Besides communication and acceptance, there are potentially other ways to deal effectively with invalidation, such as enhancement of mindfulness [50], compassion [51], and self-efficacy [49, 52]. Thus, although invalidating people is an unfair case of injustice, the invalidated person is an agent that should take a role in changing the situation.

The results of the current study should be interpreted while acknowledging that with the cross-sectional design associations can be observed without being able to draw conclusions about causal relations. Some other limitations are worth mentioning. First, a possible recruitment bias may have led to a fibromyalgia sample in which people felt more understood than the overall fibromyalgia population since the current sample consists of persons recruited in a specialized fibromyalgia clinic. Second, using the HADS, it may be difficult to distinguish anxiety from depression due to a strong common psychological distress factor [53, 54]. A third limitation is the overrepresentation of women. Strengths of the current study include the large sample size with people with fibromyalgia diagnosed by a medical professional, and, it being the first study examining both dimensions of invalidation and its relation with anxiety and depression.

Our results imply that health professionals should be alert on the level of discounting in people with fibromyalgia. Likely, discounting, fibromyalgia severity, anxiety and depression can best be considered a network model with mutually interacting factors. For each individual, it should be determined which of these is the most primary and influencing factor. Treating one or more of these factors may have positive consequences for other factors. Moreover, ongoing effort of patient associations, health policy makers, and societal organizations is needed to educate people about acknowledging the illness. Furthermore, interventions should be developed and evaluated to empower patients to deal with invalidation, e.g., by enhancing one’s communication skills, self-efficacy, acceptance, and compassion.

To conclude, this study in people with fibromyalgia shows the significance of invalidation for mental and physical health. Discounting seems the most toxic dimension of invalidation. The results suggests that overt negative responses should get attention in management of fibromyalgia, especially in people with more severe fibromyalgia.

## Electronic supplementary material

Below is the link to the electronic supplementary material.

Supplementary Material 1

## Declarations

### Open data sharing

The dataset from this study is unavailable due to a lack of participant consent for data sharing.
