# Supplementary material for: Discounting seems the most toxic dimension of invalidation in fibromyalgia: a cross-sectional analysis
Source: Rheumatol Int. 2025 Apr 15;45(5):101. doi: 10.1007/s00296-025-05850-2 (PMC12000205; doi:10.1007/s00296-025-05850-2)
Supplement: Supplementary file 1 — Supplementary Material 1 [file 296_2025_5850_MOESM1_ESM.docx]

**Supplementary Material**

**Table S1**. Pearson Correlations between the invalidation sources (discounting and lack of understanding), severity of fibromyalgia, anxiety, and depression.

|  | **1** | **2** | **3** | **4** | **5** | **6** | **7** | **8** | **9** | **10** |
| --- | --- | --- | --- | --- | --- | --- | --- | --- | --- | --- |
| 1. Discounting (3*I)^1^ spouse | **-** |  |  |  |  |  |  |  |  |  |
| 2. Discounting(3*I)^1^ family | 0.39^**^ | - |  |  |  |  |  |  |  |  |
| 3. Discounting (3*I)^1^ med. prof. | 0.38^**^ | 0.39^**^ | - |  |  |  |  |  |  |  |
| 4. Discounting (3*I)^1^ work | 0.18.^*^ | 0.37^**^ | 0.37^**^ | - |  |  |  |  |  |  |
| 5. Discounting (3*I)^1^ social serv. | 0.14 | 0.32^**^ | 0.42^**^ | 0.48^**^ | - |  |  |  |  |  |
| 6. Lack of understanding (3*I)^1^ spouse | 0.66^**^ | 0.16^*^ | 0.16^*^ | - 0.11 | 0.06 | - |  |  |  |  |
| 7. Lack of understanding (3*I)^1^ family | 0.31^**^ | 0.69^**^ | 0.24^**^ | 0.13 | 0.21^*^ | 0.38^**^ | - |  |  |  |
| 8. Lack of understanding (3*I)^1^: med. prof. | 0.27^**^ | 0.22^**^ | 0.71^**^ | 0.20^**^ | 0.25^**^ | 0.13 | 0.27^**^ | - |  |  |
| 9. Lack of understanding (3*I)^1^work | 0.10 | 0.22^**^ | 0.10 | 0.59^**^ | 0.08 | 0.26^**^ | 0.30^**^ | 0.17^**^ | - |  |
| 10. Lack of understanding (3*I)^1^ social serv. | 0.02 | 0.25^**^ | 0.25^**^ | 0.13 | 0.60^**^ | 0 .11 | 0.29^**^ | 0.32^**^ | 0.23^*^ | - |
| **11. Severity of fibromyalgia (FIQ)^2^** | **0.07** | **0.22^**^** | **0.12^*^** | **0.23^**^** | **0.33^**^** | **-0.03** | **0.05** | **0.01** | **0.18^*^** | **0.17** |
| **12. Anxiety (HADS)^3^** | **0.15^*^** | **0.23^**^** | **0.12^*^** | **0.22^**^** | **0.14** | **0.08** | **0.11** | **0.04** | **0.03** | **0.02** |
| **13. Depression (HADS)^3^** | **0 .19^**^** | **0.27^**^** | **0 .13^*^** | **0.27^**^** | **0.17^*^** | **0.10^*^** | **0.24^**^** | **0.02** | **0.15^*^** | **0.01** |

^*^*p* <.05; ^**^ *p* <.01

^1^ = 3*I: Illness Invalidation Inventory; ^2^ = FIQ: Fibromyalgia Impact Questionnaire; ^3^ = HADS: Hospital Anxiety and Depression Scale
